# Gas6/Axl-mediated macrophage hyporesponsiveness drives severe pulmonary mucormycosis in diabetic mice

**DOI:** 10.3389/fcimb.2026.1939483

**Published:** 2026-09-07

**Authors:** Shotaro Kondo, Keito Owaki, Gento Hamasaki, Tatsuya Inukai, Jotaro Kinoshita, Toshihiro Ito, Shigeki Nakamura, Takehiko Shibata

**Affiliations:** 1 Department of Microbiology, Tokyo Medical University, Tokyo, Japan; 2 Department of Immunology, Nara Medical University, Nara, Japan

**Keywords:** GAS6/AXL, innate immune paralysis, mucormycosis, neutrophil recruitment, type 1 diabetes

## Abstract

**Background:**

Pulmonary mucormycosis is a life-threatening fungal infection with a mortality rate exceeding 50%, and diabetes mellitus is one of its strongest risk factors. However, the molecular mechanisms linking diabetes to impaired pulmonary innate immunity remain poorly understood.

**Methods:**

A streptozotocin-induced type 1 diabetes (T1D) mouse model was used to investigate host responses to intratracheal *Cunninghamella bertholletiae* infection. Genetic Axl deficiency, pharmacological Axl inhibition with BGB324, histopathological analyses, and ex vivo and *in vitro* macrophage assays were performed to define the role of the Gas6/Axl axis in antifungal immunity.

**Results:**

T1D mice exhibited rapid mortality after pulmonary *C. bertholletiae* infection, accompanied by markedly impaired early neutrophil recruitment. Elevated lung and serum levels of growth arrest-specific protein 6 (Gas6) were intrinsic features of the diabetic state and correlated with blood glucose levels. Genetic ablation or pharmacological blockade of Axl restored macrophage chemokine responses, increased neutrophil recruitment into the airspace, reduced fungal burden, and significantly improved survival in T1D mice. Mechanistically, the Gas6/Axl axis directly suppressed fungus-induced chemokine production by alveolar macrophages, establishing a pre-existing state of innate immune hyporesponsiveness before infection.

**Conclusions:**

These findings identify the Gas6/Axl axis as a mechanistic link between diabetes and impaired pulmonary innate immunity. Targeting this pathway may represent a promising host-directed therapeutic strategy for pulmonary mucormycosis in patients with diabetes.

## Introduction

Diabetes mellitus is a major global metabolic disorder that profoundly alters host immune responses beyond glycemic dysregulation. The coronavirus disease 2019 (COVID-19) pandemic highlighted this vulnerability, identifying diabetes and obesity as key determinants of disease severity and mortality (Ando et al., 2021; Zhou et al., 2021). These observations suggest that metabolic dysfunction broadly reshapes innate immunity and increases susceptibility to severe infectious diseases.

Besides viral infections, diabetes represents the most common risk factor for invasive mucormycosis worldwide and accounts for a substantial proportion of cases, particularly in Asia (Skiada et al., 2020; Roden et al., 2005). Despite advances in antifungal therapy and surgical management, mortality remains high, especially in disseminated disease (Roden et al., 2005; Ibrahim et al., 2012). Among Mucorales species, *Cunninghamella bertholletiae* (*C. bertholletiae*) is associated with particularly severe outcomes. However, the mechanisms by which diabetes predisposes to mucormycosis remain incompletely understood.

Effective host defense against Mucorales critically depends on rapid neutrophil recruitment to infected tissues, a process orchestrated by macrophage-derived chemokines, including CXCL1 and CXCL2 (Roilides et al., 2012; Drummond et al., 2015). Impaired neutrophil responses are strongly associated with poor outcomes in mucormycosis (Roilides et al., 2012). Although hyperglycemia disrupts multiple components of innate immunity, including chemokine production and neutrophil chemotaxis, the upstream pathways responsible for impaired early antifungal responses in the diabetic lung remain unclear (Drummond et al., 2015; Delamaire et al., 1997; Geerlings and Hoepelman, 1999).

Growth arrest-specific protein 6 (Gas6) is a vitamin K–dependent ligand of the TAM receptor family, including Axl, and functions as a negative regulator of innate immune activation (Lemke, 2013). Gas6/Axl signaling suppresses inflammatory responses in macrophages and dendritic cells, partly through induction of suppressor of cytokine signaling (SOCS) proteins (Lemke, 2013). Previous studies from our group demonstrated that this pathway dampens antiviral and antimicrobial immunity during infection; thereby, promoting pathogen persistence (Shibata et al., 2014b; Shibata et al., 2020, Shibata et al., 2014c). Emerging evidence from both clinical and preclinical studies reveals that the Gas6/Axl axis functions as a central regulatory hub in antiviral immunity, where dysregulation of this pathway predicts severity and mortality across diverse respiratory viral infections (Morales et al., 2021; Shibata et al., 2020; Lemke, 2013; Rothlin et al., 2007). Particularly, excessive Gas6 activation in tissue macrophages suppresses direct antiviral responses and simultaneously establishes a permissive microenvironment for secondary pathogen invasion (Shibata et al., 2020). Despite the clear clinical relevance of Gas6 dysregulation in infectious diseases, its contribution to fungal pathogenesis, especially in the setting of metabolic disorders such as diabetes mellitus, remains entirely unexplored.

Given associations between elevated Gas6 signaling and immune suppression in other infectious settings, the involvement of this pathway in impaired host defense within the diabetic lung appeared plausible. To address this hypothesis, a streptozotocin (STZ)-induced type 1 diabetes mouse model challenged with *C. bertholletiae* was employed in combination with genetic and pharmacological modulation of the Gas6/Axl axis.

## Methods

### Sex as a biological variable

Only male mice were used in this study because streptozotocin-induced type 1 diabetes is more consistently induced in males than in females, enabling stable and reproducible evaluation of infection severity and immune responses. In addition, the use of male mice minimized variability associated with hormonal cycling. Whether the mechanisms identified in this study extend to female mice remains unknown and requires future investigation.

### Mice and STZ-induced diabetes model

Specific pathogen-free, 6-week-old male C57BL/6J mice were purchased from Japan SLC, Inc. (Hamamatsu, Japan). Axl^-/-^ mice were generously provided by Dr. Carla Rothlin (Yale University, New Haven, CT). All Axl^-/-^ mice used in this study were backcrossed onto the C57BL/6J background for ≥10 generations. To induce T1D, mice received intraperitoneal injections of STZ (FUJIFILM Wako Pure Chemical Corporation, Osaka, Japan) at a dose of 200 mg/kg. Blood glucose levels were measured using a glucose meter (FORA GD40; ForaCare Japan Co., Ltd., Tokyo, Japan), and mice with non-fasting blood glucose levels exceeding 300 mg/dL were considered diabetic and used for subsequent inoculation experiments. The maximum detectable limit of the glucose meter was 600 mg/dL. All animal experiments were performed according to the guidelines of the Committees on Animal Handling and Ethical Regulations of Tokyo Medical University, Japan. The research protocol was approved by the Institutional Animal Care and Use Committee of the institute (R6-046, R7-011).

### Fungal strain and sporangiospore preparation

The clinical isolate of Cb used in this study was originally isolated from a patient and was kindly provided by Dr. Yoshitsugu Miyazaki (National Institute of Infectious Diseases, Japan Institute for Health Security, Tokyo, Japan). Cb was cultured on potato dextrose agar (PDA) at 37 °C for 7 days. Spores were harvested by flushing the agar surface with sterile phosphate-buffered saline (PBS) containing 0.05% Tween 80, as previously described with minor modifications (Inukai et al., 2025). The spore suspension was filtered through a 40-μm EASY strainer (Greiner Bio-One Co. Ltd., Tokyo, Japan) to remove hyphal fragments and was subsequently washed twice with PBS. Spore concentrations were determined using a hemocytometer and adjusted to the required density before inoculation.

### Pulmonary inoculation model

Mice were anesthetized via intraperitoneal (i.p.) injection of a three-drug combination (MMB) consisting of 0.3 mg/kg medetomidine hydrochloride, 4.0 mg/kg midazolam, and 5.0 mg/kg butorphanol tartrate. Sufficient depth of anesthesia was confirmed by the loss of the pedal withdrawal reflex before the procedure. The fungal challenge was performed using an oropharyngeal aspiration technique. Briefly, anesthetized mice were placed in a supine position with the upper body slightly elevated. The tongue was gently extended forward, and 50 μL of the Cb suspension (or PBS for control groups) was applied to the tongue base. The nostrils were briefly occluded to induce aspiration of the liquid into the lungs. After confirming normal thoracic movement, mice were monitored until they fully recovered from anesthesia. Survival was monitored daily for 7 days. For tissue analysis, mice were deeply anesthetized with isoflurane and euthanized by cervical dislocation at 24 h post-inoculation. Lung tissues, BAL fluid, and serum samples were then collected.

### BAL cell analysis

BAL fluid was collected with sterile PBS, and the total number of recovered cells was determined. BAL cells were then cytospun onto glass slides and stained with Diff-Quik. Differential cell counting was performed based on cellular morphology, and neutrophils were identified by their characteristic multilobed nuclei. The percentage of neutrophils was multiplied by the total BAL cell count to calculate the absolute number of neutrophils recovered from each mouse.

### Pharmacological inhibition of Axl

For Axl inhibition, the small-molecule Axl inhibitor BGB324 (Abcam, Cambridge, UK) was administered to diabetic mice. BGB324 was dissolved in 0.5% hydroxypropyl methylcellulose (Alfa Aesar, MA) and administered intraperitoneally at a dose of 250 μg/mouse once daily for 7 days before fungal inoculation. Control mice received the vehicle alone.

### Ex vivo alveolar macrophage stimulation

AMs were collected from BAL fluid obtained from naïve control and T1D mice using sterile PBS. Collected cells were centrifuged at 400 × g for 5 min and resuspended in Roswell Park Memorial Institute (RPMI) 1640 medium supplemented with 10% fetal bovine serum (FBS). AMs were seeded at a density of 2 × 10^5^ cells/well in 24-well plates and allowed to adhere for 2 h at 37 °C. After washing away non-adherent cells, AMs were co-cultured with Cb spores at a multiplicity of infection (MOI) = 10:1. At 6 h post-inoculation, total RNA was extracted for quantitative reverse transcription-polymerase chain reaction (RT-PCR) analysis of CXCL1 and CXCL2 expression. At 24 h post-inoculation, the culture supernatants were collected for measurement of CXCL1 and CXCL2 protein concentrations. CXCL1 and CXCL2 protein concentrations were measured using specific enzyme-linked immunosorbent assay (ELISA) kits (R&D Systems, Minneapolis, MN) according to the manufacturer’s instructions.

### *In vitro* macrophage treatment

AMs were isolated from the BAL fluid collected from naïve (normal) mice. Cells were seeded in 48-well plates at a density of 2 × 10^5^ cells/well in RPMI 1640 medium supplemented with 10% FBS. After 2 h of incubation to allow cell attachment, macrophages were stimulated with rGas6 (100 ng/mL) for 24 h. Cells were then co-cultured with Cb spores at an MOI = 10:1 for 6 h. Cell lysates were collected at the indicated time points for gene expression analysis. The culture supernatants were collected for measurement of CXCL1 and CXCL2 protein concentrations at 24 h post-inoculation.

### Quantification of cytokines and chemokines

Total RNA was extracted from lung tissues or cultured cells using TRIzol reagent (Invitrogen/Life Technologies, Carlsbad, CA). Complementary deoxyribonucleic acid (cDNA) was synthesized using TaqMan reverse transcription reagents (Applied Biosystems, Foster City, CA). Quantitative PCR was then performed on a StepOnePlus Real-Time PCR System (Applied Biosystems) using TaqMan Fast Advanced Master Mix (Applied Biosystems). Target gene expression was quantified using TaqMan Gene Expression Assays (Applied Biosystems) with specific primers and probes for CXCL1 and CXCL2. Relative expression levels were normalized to *Gapdh* as an internal control using the ΔΔCt method. Protein concentrations in BAL fluid or serum were measured using specific ELISA kits according to the manufacturer’s instructions. The ELISA detection limits for the cytokines were as follows: Gas6, >39.1 pg/mL; CXCL1, >15.6 pg/mL; CXCL2, >15.6 pg/mL.

### Histological and immunohistochemical analysis

Lung tissues were fully inflated with 10% neutral buffered formalin, dissected, and fixed in fresh 10% formalin at room temperature for 24 h. Following fixation, tissues were embedded in paraffin using standard histological techniques and sectioned at 5 μm thickness. For structural evaluation and fungal detection, sections were stained with H&E and GMS, respectively. For Gas6 detection, rehydrated sections were incubated with a goat anti-mouse Gas6 polyclonal antibody (10 μg/mL; AF986-SP, R&D Systems). Control tissue sections were incubated with a normal goat immunoglobulin G (IgG) isotype control antibody (10 μg/mL; I-5000, Vector Laboratories). The slides were then developed using the ImmPRESS HRP Horse Anti-Goat IgG Polymer Detection Kit (Peroxidase) and 3,3’-diaminobenzidine (DAB) (Vector Laboratories), following the manufacturer’s instructions. Low- and high-magnification images were captured using a light microscope (OLYMPUS BX51, Tokyo, Japan). Histological and immunohistochemical quantification was performed using Fiji software (ImageJ). To assess the fungal burden, three representative fields were randomly selected from the GMS-stained sections of the left lung from each mouse. The fungal burden was calculated as the ratio of the GMS-positive hyphal area to the total lung tissue area (identified by light green counterstaining), and the mean value from the three fields was used for statistical analysis, as previously described (Stolz et al., 2018).

### Statistical analysis

Data were represented as mean ± standard error of the mean. Statistical significance was determined using the Mann–Whitney test for two-group comparisons. The correlation between blood glucose levels and serum Gas6 concentrations was analyzed using Pearson’s correlation coefficient. Survival curves were analyzed using the log-rank (Mantel-Cox) test. Statistical significance was set at P < 0.05.

## Results

### T1D mice succumb to pulmonary mucormycosis in association with impaired neutrophil recruitment into the airspace

**Figure 1:**
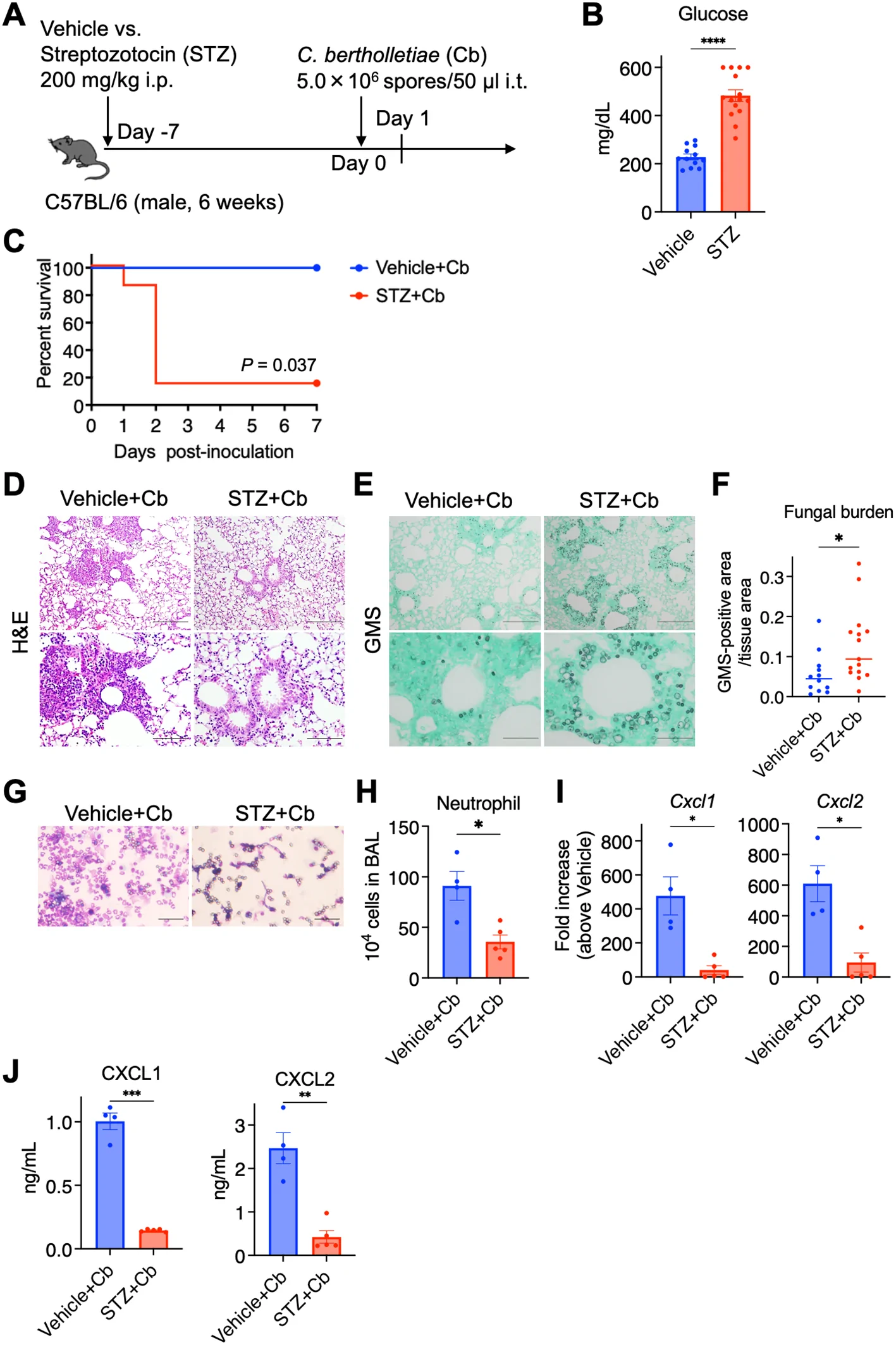
T1D mice succumb to pulmonary mucormycosis in association with impaired neutrophil recruitment into the airspace. **(A)** Experimental scheme for the induction of type 1 diabetes (T1D) using streptozotocin (STZ) and subsequent *Cunninghamella bertholletiae* (Cb) inoculation. **(B)** Non-fasting blood glucose levels in vehicle-treated control mice (n = 12 mice per group) and STZ-induced T1D mice (n = 15 mice per group) were measured before inoculation. **(C)** Survival of vehicle-treated control mice and STZ-induced T1D mice (n = 10 mice per group) following intratracheal inoculation with Cb (5.0 × 10^6^ spores). **(D)** Representative images of Hematoxylin and Eosin (H&E)-stained lung sections at 24 h post-inoculation. **(E)** Representative images of Grocott’s Methenamine Silver (GMS)-stained lung sections at 24 h post-inoculation. Scale bars: 50 μm (upper images); 100 μm (lower images). **(F)** Quantification of GMS-positive areas in lung sections. For each mouse, the percentage of GMS-positive area was calculated as the average of three non-overlapping fields of view (n = 5 mice per group). **(G)** Representative images of cytospin preparations of bronchoalveolar lavage (BAL) fluid from control and T1D mice at 24 h post-inoculation. Neutrophils were identified by their characteristic multi-lobed nuclei in Diff-Quik–stained samples. Scale bars: 50 μm. **(H)** Absolute numbers of neutrophils in BAL fluid, determined by total cell counts and differential counting of cytospin samples (n = 5 mice per group). **(I)** Messenger ribonucleic acid (mRNA) expression levels of CXCL1 and CXCL2 in lung tissues at 24 h post-Cb inoculation. **(J)** Protein concentrations of CXCL1 and CXCL2 in BAL fluid at 24 h post-Cb inoculation, measured by enzyme-linked immunosorbent assay (ELISA).

To investigate the impact of diabetes on host defense against mucormycosis, a STZ-induced type 1 diabetes (T1D) mouse model was employed (**Figure 1A**). STZ-treated mice developed severe and sustained hyperglycemia, as evidenced by non-fasting blood glucose levels exceeding 300 mg/dL 1 week after injection (Wu and Huan, 2008; Graham et al., 2011), whereas vehicle-treated control mice remained normoglycemic (**Figure 1B**). Following intratracheal inoculation with *C. bertholletiae* (Cb), T1D mice exhibited a rapid and progressive mortality, reaching 82% within 48 h post-inoculation (**Figure 1C**). In contrast, all normoglycemic control mice survived the fungal challenge.

Histological assessment of lung tissues was performed to evaluate inflammation and fungal invasion. Hematoxylin and eosin (H&E) staining revealed increased inflammatory cell accumulation in the alveolar and peribronchiolar spaces in control mice compared with T1D mice (**Figure 1D**). This suggests that the diabetic environment suppresses the initial inflammatory signaling required to orchestrate an effective host defense against Cb.

In contrast, despite the relatively attenuated inflammatory appearance in the lungs of T1D mice, Grocott’s methenamine silver (GMS) staining at 24 h post-inoculation revealed extensive and invasive fungal hyphal growth throughout the lung parenchyma (**Figure 1E**). Morphometric quantification, calculated as the ratio of the GMS-positive fungal area to the total lung tissue area, confirmed that the fungal burden was significantly higher in T1D mice than in controls (**Figure 1F**). These findings demonstrate that the diabetic state results in a profound failure to control Cb proliferation, characterized by a defective inflammatory response and uncontrolled fungal overgrowth.

### Neutrophils are indispensable for protective immunity against *C. bertholletiae*

To confirm the essential role of neutrophils in our pulmonary mucormycosis model, neutrophils were depleted using an anti-Ly6G monoclonal antibody before Cb inoculation in non-diabetic mice (**Supplementary Figure S1A**). Neutrophil-depleted mice succumbed to Cb infection within 4 days, whereas control IgG-treated mice survived, demonstrating an essential role for neutrophils in host survival (**Supplementary Figure S1B**). Analysis of the bronchoalveolar lavage (BAL) fluid and lung tissues at 24 h post-inoculation further supported these findings. In control IgG-treated mice, robust neutrophil recruitment was observed in the BAL fluid and was accompanied by extracellular deoxyribonucleic acid (DNA)-like structures suggestive of neutrophil extracellular traps (NETs) (**Supplementary Figure S1C**, left). In contrast, anti-Ly6G-treated mice showed a near-complete absence of neutrophil recruitment in the BAL fluid, permitting Cb germination and extensive fungal hyphal formation (**Supplementary Figure S1C**, right). Quantification of BAL cells confirmed a marked reduction in the absolute number of neutrophils in the BAL fluid from anti-Ly6G-treated mice (**Supplementary Figure S1D**). GMS staining of lung sections confirmed extensive fungal germination and hyphal development in the absence of neutrophils (**Supplementary Figure S1E**). Quantitative analysis further validated a significantly higher fungal burden in neutrophil-depleted mice compared with control mice (**Supplementary Figure S1F**). These findings demonstrate the functional requirement of neutrophils for host defense against Cb in the infected airspace.

### Impaired early neutrophil recruitment and chemokine production in diabetic hosts

To further characterize the immune defect, neutrophil recruitment during the early phase of inoculation (24 h) was evaluated. Cytospin-based differential counting of BAL cells revealed a significant reduction in the absolute number of neutrophils recovered from the airspace of T1D mice (**Figures 1G, H**). Absolute neutrophil numbers were calculated from total BAL cell counts and differential counting of Diff-Quik-stained cytospin preparations. Notably, the STZ+Cb group exhibited sparse neutrophil distribution accompanied by elongated Mucorales hyphae, a feature absent in the control group. This defect in neutrophil recruitment was associated with markedly reduced CXCL1 and CXCL2 expression in T1D mice at 24 h post-inoculation. CXCL1 and CXCL2 mRNA levels were reduced in lung tissues, and their protein concentrations were also decreased in BAL fluid (**Figures 1I, J**).

### Elevated Gas6 expression in the airway epithelium of diabetic mice

**Figure 2:**
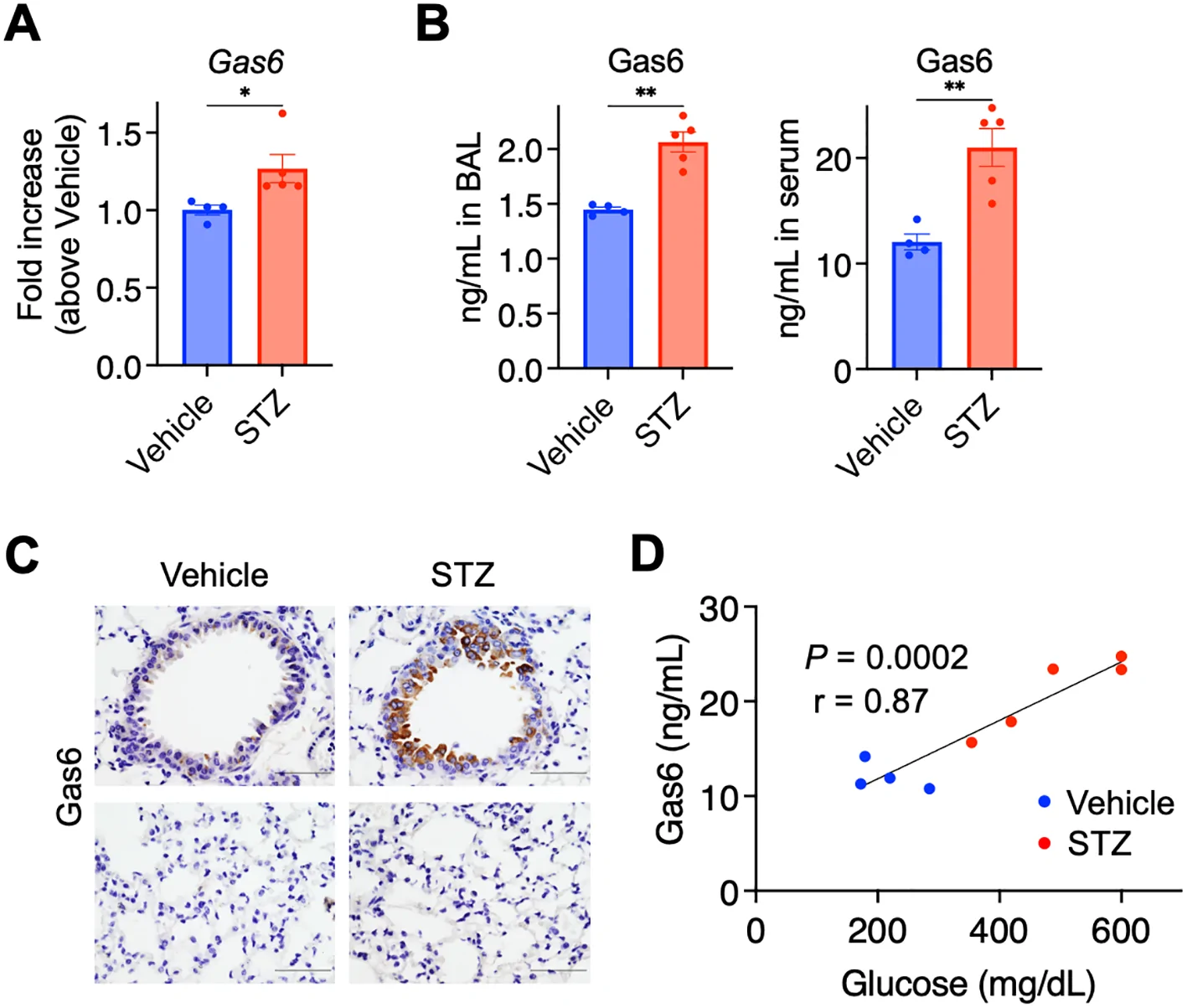
Basal induction of Gas6 in T1D mice. **(A)** Relative messenger ribonucleic acid (mRNA) expression of growth arrest-specific protein 6 (Gas6) in homogenized lung tissues from vehicle-treated control mice and streptozotocin (STZ)-induced type 1 diabetes (T1D) mice (n = 5 mice per group), as determined by quantitative reverse transcription-polymerase chain reaction. **(B)** Gas6 protein concentrations in bronchoalveolar lavage fluid (left) and serum (right) from control and T1D mice measured by enzyme-linked immunosorbent assay (n = 5 mice per group). Data are representative of three independent experiments with similar results and are presented as mean ± standard error of the mean. *P < 0.05, **P < 0.01 by Mann–Whitney test. **(C)** Representative immunohistochemistry staining for Gas6 in lung sections. Arrows indicate Gas6-positive signals in airway epithelial cells. Scale bars: 100 μm. **(D)** Correlation between blood glucose and serum Gas6 levels. Scatter plot showing the correlation between blood glucose levels and serum Gas6 concentrations across control and T1D mice. The solid line represents the linear regression. Statistical significance was determined by Pearson’s correlation coefficient (r = 0.87, P = 0.0002).

Potential inhibitory pathways responsible for the suppressed chemokine response in T1D hosts were next investigated. The Gas6/Axl axis is a known suppressor of macrophage-mediated innate immunity in certain infection models (Shibata et al., 2020; Ishikawa et al., 2026). Based on these observations, the involvement of this pathway in diabetic lung immune dysfunction was examined. Even in the absence of infection, Gas6 mRNA expression was significantly upregulated in the lungs of T1D mice compared with controls (**Figure 2A**). This was accompanied by a significant increase in Gas6 protein levels in both BAL fluid and serum of T1D mice (**Figure 2B**). Notably, immunohistochemical analysis revealed that Gas6 was predominantly and intensely expressed in the airway epithelial cells of T1D mice (**Figure 2C**). Although faint Gas6 signals were occasionally observed in alveolar epithelial cells and macrophages, they were markedly less prominent than the robust expression seen in the airway epithelium. These findings suggest that the diabetic environment induces aberrant Gas6 production, particularly from the airway epithelium, potentially creating an inhibitory pulmonary milieu.

Intriguingly, a strong positive correlation was observed between blood glucose levels and serum Gas6 concentrations (**Figure 2D**). This robust association suggests that the systemic elevation of Gas6 is closely linked to hyperglycemia severity in T1D mice, potentially serving as a pre-existing factor that modulates the host immune environment before fungal exposure.

### Axl deficiency restores innate host defense and limits fungal proliferation in T1D mice

Given the marked elevation of Gas6 expression in lungs from T1D mice (**Figure 2**), the contribution of downstream signaling through its cognate receptor was next investigated, with particular focus on Axl, a high-affinity receptor for Gas6.

**Figure 3:**
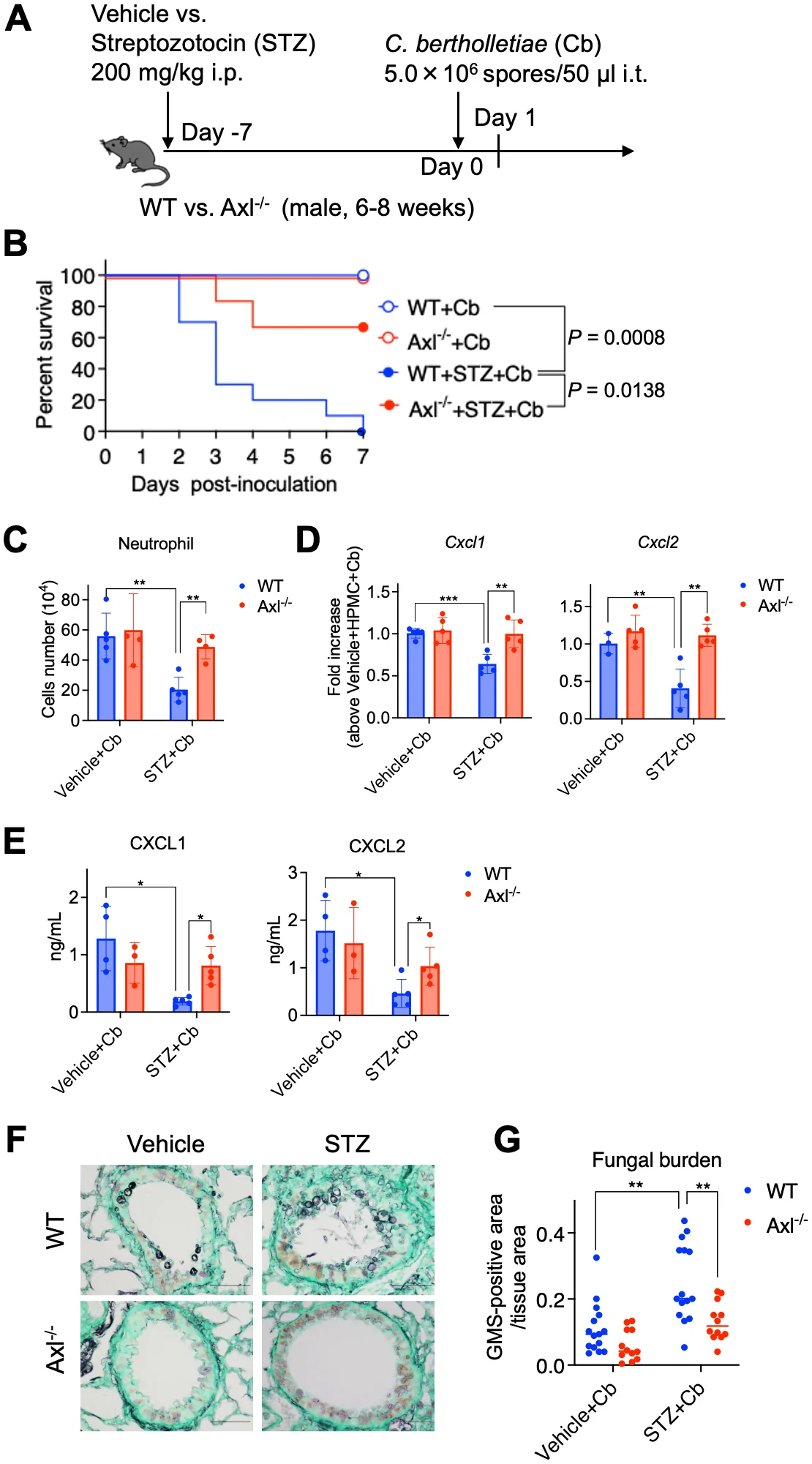
Genetic deletion of Axl rescues T1D mice from lethal Cb inoculation. **(A)** Experimental scheme for the induction of type 1 diabetes (T1D) using streptozotocin (STZ) and subsequent *Cunninghamella bertholletiae* (Cb) inoculation in wild-type (WT) and *Axl*-deficient (Axl^−/−^) mice. **(B)** Survival of T1D WT and T1D Axl^−/−^ mice after i.t. inoculation with Cb (5.0 × 10^6^ spores). P = 0.037 by log-rank test. **(C)** Absolute number of neutrophils recovered in BAL fluid at 24 h post-inoculation. **(D)** Messenger ribonucleic acid (mRNA) expression levels of CXCL1 and CXCL2 in lung tissues at 24 h post-Cb inoculation. **(E)** Protein concentrations of CXCL1 and CXCL2 in BAL fluid at 24 h post-Cb inoculation, measured by ELISA. **(F)** Representative images of Grocott’s Methenamine Silver (GMS)-stained lung sections at 24 h post-inoculation. Scale bars: 200 μm. **(G)** Quantification of GMS-positive areas in lung sections. For each mouse, the percentage of GMS-positive area was calculated as the average of three non-overlapping fields of view (n = 5 mice per group). Data are representative of two independent experiments with similar results and are presented as mean ± standard error of the mean. *P < 0.05, **P < 0.01 by Mann–Whitney test.

Axl-deficient (Axl^−/−^) mice were employed to determine the functional contribution of Gas6/Axl signaling to increased susceptibility to pulmonary mucormycosis in T1D mice (**Figure 3A**). Although T1D wild-type (WT) mice rapidly succumbed within 48 h, T1D Axl^−/−^ mice showed a significantly improved survival rate (**Figure 3B**). Consistent with the observed phenotype in diabetic mice, T1D Axl−/− mice displayed a robust restoration of neutrophil recruitment into the lungs (**Figure 3C**), accompanied by restored CXCL1 and CXCL2 production. CXCL1 and CXCL2 mRNA levels were increased in lung tissues (**Figure 3D**), and their protein concentrations in BAL fluid were also restored in T1D Axl^−/−^ mice (**Figure 3E**). Histopathological analysis further confirmed the protective effect associated with Axl deficiency. T1D WT mice showed extensive fungal hyphal invasion and severe tissue destruction, whereas such pathological changes were markedly attenuated in T1D Axl^−/−^ mice (**Figures 3F, G**). Consistent with these observations, the fungal burden, quantified as GMS-positive area, was significantly reduced in lungs from T1D Axl^−/−^ mice. Collectively, these results provide strong genetic evidence that signaling through the Gas6 receptor Axl constitutes a major driver of immune dysfunction and fungal overgrowth in the diabetic lung.

### Pharmacological Axl blockade by BGB324 rescues T1D mice from lethal pulmonary mucormycosis

**Figure 4:**
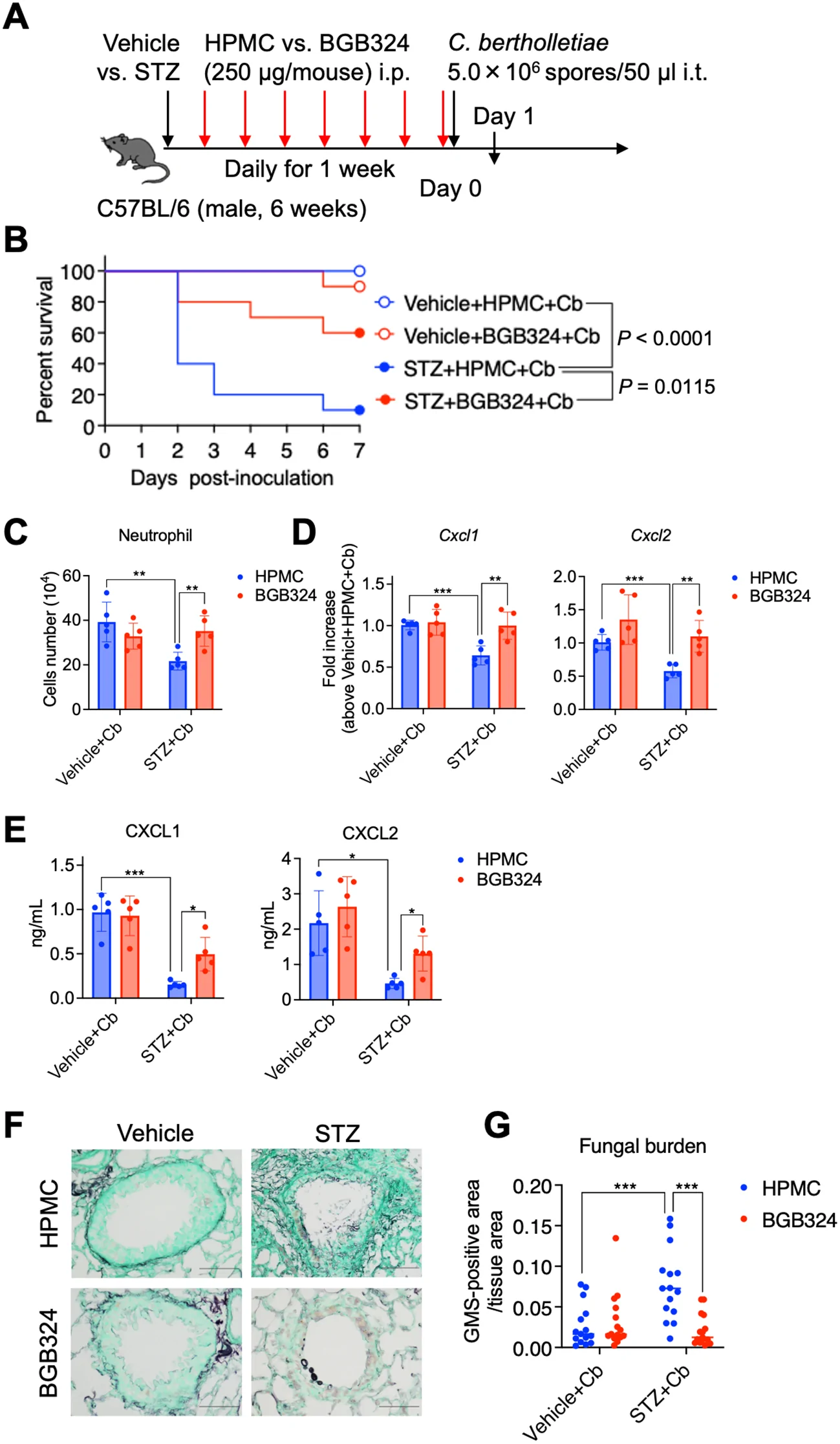
Pharmacological blockade of Axl restores macrophage function and innate immunity. **(A)** Experimental scheme for BGB324 treatment (250 μg/mouse, daily). **(B)** Survival of type 1 diabetes (T1D) mice treated with hydroxypropyl methylcellulose (HPMC; vehicle) or BGB324 after *Cunninghamella bertholletiae* **(Cb)** inoculation (n = 10 mice per group). **(C)** Absolute number of neutrophils in bronchoalveolar lavage fluid at 24 h post-inoculation. **(D)** Messenger ribonucleic acid (mRNA) expression levels of CXCL1 and CXCL2 in lung tissues at 24 h post-Cb inoculation. **(E)** Protein concentrations of CXCL1 and CXCL2 in BAL fluid at 24 h post-Cb inoculation, measured by ELISA. **(F)** Representative images of GMS-stained lung sections at 24 h post-inoculation. Scale bars: 200 μm. **(G)** Quantification of Grocott’s Methenamine Silver (GMS)-positive areas in lung sections. For each mouse, the percentage of GMS-positive area was calculated as the average of three non-overlapping fields of view (n = 5 mice per group). Data are representative of two independent experiments with similar results and are presented as mean ± standard error of the mean. **P < 0.01, ***P < 0.001 by Mann–Whitney test.

To explore the therapeutic potential of targeting the Axl pathway, BGB324, a selective small-molecule inhibitor, was administered to T1D mice beginning 1 week before Cb inoculation (**Figure 4A**). Notably, pharmacological intervention significantly prolonged survival in T1D mice (**Figure 4B**) and effectively restored neutrophil recruitment into the airspace (**Figure 4C**). This improvement in neutrophil recruitment was accompanied by increased CXCL1 and CXCL2 expression. CXCL1 and CXCL2 mRNA levels were increased in lung tissues, while their protein concentrations in BAL fluid were also restored following BGB324 treatment (**Figures 4D, E**). Although vehicle-treated T1D mice exhibited extensive fungal hyphal invasion and severe tissue destruction, these pathological hallmarks were markedly attenuated in BGB324-treated T1D mice (**Figures 4F, G**).

Collectively, these results from both genetic ablation (**Figure 3**) and pharmacological inhibition (**Figure 4**) provide convergent evidence that the Gas6/Axl axis is a major driver of innate immune dysfunction in the diabetic lung. These findings underscore that targeting the Gas6/Axl axis elucidates the molecular basis underlying immune hyporesponsiveness in diabetes and represents a potential host-directed therapeutic strategy against lethal mucormycosis.

Because neutrophils express minimal Axl, whereas alveolar macrophages (AMs) express high levels of this receptor (Shibata et al., 2020), and considering that AMs constitute a major source of neutrophil-attracting chemokines such as CXCL1 and CXCL2, the restoration of antifungal immunity following Axl inhibition was hypothesized to result from functional reprogramming of AMs. Therefore, subsequent experiments focused on AMs to define mechanisms through which the Gas6/Axl axis regulates macrophage function.

### The Gas6/Axl axis directly mediates the functional impairment of diabetic alveolar macrophages

**Figure 5:**
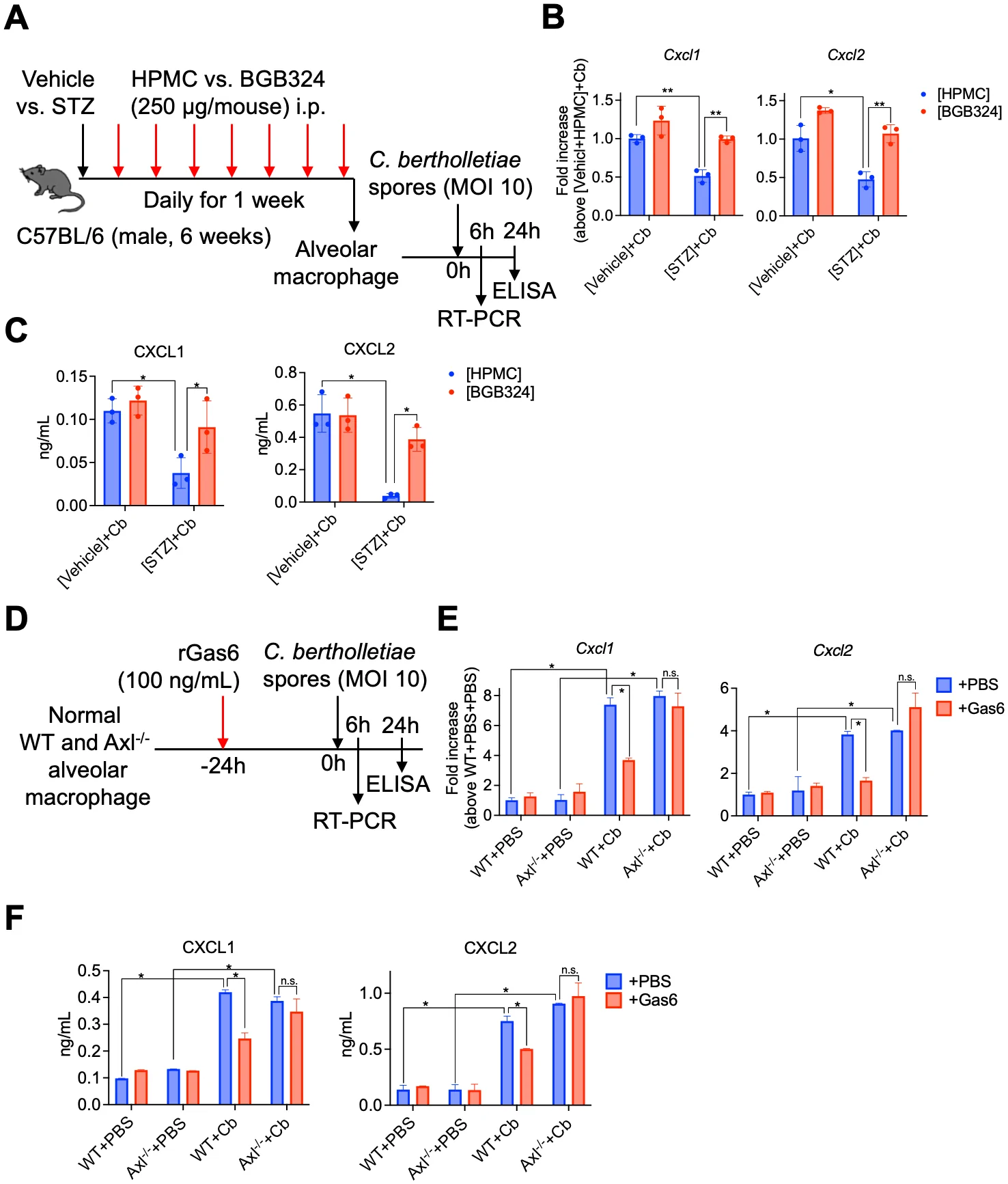
Gas6/Axl directly suppresses macrophage-derived chemokine production. **(A)** Alveolar macrophages (AMs) were isolated from non-diabetic control mice or type 1 diabetes (T1D) mice and stimulated with *Cunninghamella bertholletiae* (Cb) spores (multiplicity of infection [MOI] = 10:1) for 6 h ex vivo. Messenger ribonucleic acid (mRNA) expression **(B)** and protein **(C)** levels of CXCL1 and CXCL2 were determined by quantitative polymerase chain reaction. **(D)** Normal AMs isolated from wild-type and Axl^-/-^ mice were pre-treated with recombinant growth arrest-specific protein 6 (100 ng/mL) for 24 h, followed by *Cb* inoculation (MOI = 10:1). mRNA expression **(E)** and protein **(F)** levels of CXCL1 and CXCL2 were measured at 6 h post-inoculation. Data are presented as mean ± standard error of the mean. *P < 0.05, **P < 0.01 by Mann–Whitney test **(B, C, E, F)**. Data are representative of two independent experiments with similar results.

To validate this hypothesis, ex vivo stimulation assays were first performed using AMs harvested from T1D mice treated with either BGB324 or vehicle (**Figure 5A**). Consistent with *in vivo* observations, AMs from vehicle-treated T1D mice exhibited a severely blunted induction of CXCL1 and CXCL2 mRNA expression and protein secretion following Cb spore stimulation (**Figures 5B, C**). In contrast, AMs from BGB324-treated T1D mice showed a remarkable restoration of these chemokine responses. These findings indicate that macrophage Axl signaling plays a major role in suppressing AM activation within the diabetic environment and that disrupting this Gas6/Axl axis is sufficient to restore AM function.

To further define the molecular basis underlying Gas6-mediated suppression, recombinant Gas6 (rGas6) was administered to AMs isolated from non-diabetic control mice before fungal stimulation (**Figure 5D**). Exogenous Gas6 significantly suppressed Cb-induced CXCL1 and CXCL2 production in WT AMs at both the mRNA and protein levels (**Figures 5E, F**). Critically, this Gas6-mediated suppression was absent in AMs derived from Axl-deficient mice, demonstrating dependence on Axl signaling. Specifically, rGas6 treatment failed to reduce CXCL1 and CXCL2 expression in Axl^−/−^ AMs following Cb stimulation; thereby, establishing Axl as the essential receptor mediating the inhibitory activity of Gas6.

Collectively, these findings demonstrate that the Gas6/Axl axis functions as a direct molecular checkpoint that restrains AM activation and impairs initiation of chemokine signaling required for neutrophil mobilization. Furthermore, the pharmacological inhibition of Axl effectively reverses this state of macrophage dysfunction and restores protective innate immune responses within the diabetic lung.

## Discussion

In this study, a mechanism underlying the heightened susceptibility of T1D hosts to invasive mucormycosis was elucidated. Elevated Gas6 drove AMs into a hyporesponsive state through Axl signaling, thereby impairing CXCL1 and CXCL2 production. Both genetic ablation of Axl and pharmacological inhibition with BGB324 restored chemokine responses and neutrophil recruitment, reducing fungal burden in diabetic mice. Importantly, both genetic deficiency of Axl and pharmacological inhibition with BGB324 improved host defense despite the diabetic background, supporting a mechanistic contribution of the Gas6/Axl pathway to the phenotype observed in this model. These findings do not completely exclude additional effects of STZ, but indicate that the impaired host defense phenotype cannot be attributed solely to STZ exposure. These convergent findings reveal an important distinction: impaired host defense in diabetes involves an actively regulated and therapeutically targetable signaling pathway rather than solely a passive metabolic consequence.

Historically, the increased susceptibility to fungal infections in patients with diabetes has been attributed to non-specific factors, including enhanced fungal growth in hyperglycemic and ketoacidotic environments, increased iron availability, and generalized impairment of neutrophil functions such as phagocytosis and chemotaxis (Delamaire et al., 1997; Dowey et al., 2021; Trevelin et al., 2017). Consistent with these concepts, impaired neutrophil recruitment during experimental *C. bertholletiae* infection has previously been reported in diabetic rabbits, although the molecular basis for this defect remained unknown (Sheldon and Bauer, 1959). Although such metabolic abnormalities undoubtedly contribute to fungal susceptibility, the present findings support an additional layer of immune dysregulation. In the current model, resident immune cells, specifically AMs, remained present within the lung but exhibited profound functional paralysis. Such dysfunction resulted in a failure to initiate chemokine signaling necessary for timely neutrophil recruitment. Critically, neutrophils entering the diabetic lung encounter both delayed recruitment and functional impairment (Delamaire et al., 1997; Dowey et al., 2021; Trevelin et al., 2017); thereby, creating a “double hit” scenario that compromises early antifungal defenses. Importantly, the restoration of macrophage chemokine responses through Axl blockade enhanced neutrophil recruitment and promoted effective fungal control (**Figure 4**), suggesting that recruitment-competent neutrophils retain substantial antimicrobial activity when appropriately mobilized.

Beyond macrophage-derived chemokines, other innate immune pathways likely contribute to antifungal defense. For instance, interleukin-17A (IL-17A), primarily produced by Th17 cells and group 3 innate lymphoid cells (ILC3), plays an important role in fungal control by promoting neutrophil mobilization and enhancing antimicrobial functions (Conti and Gaffen, 2015; Gladiator and LeibundGut-Landmann, 2013). However, the rapid kinetics in the current infection model with T1D mice exhibiting 82% mortality within 48 h, indicating that early macrophage-dependent chemokine responses constitute a rate-limiting step before full adaptive immune mobilization. This timing-dependent vulnerability highlights why the macrophage-mediated Gas6/Axl axis emerges as a critical control point in the immediate post-infection phase.

Notably, we previously reported that the Gas6/Axl axis acts as a potent suppressor of pulmonary innate immunity in models of invasive pulmonary aspergillosis (IPA), where neutrophil depletion was required to achieve lethal infection (Shibata et al., 2014a). In the present study, the diabetic environment itself generated a sufficient “pre-activated” Gas6/Axl signal to induce a similar state of immune suppression. Such preconditioning enabled Cb, a highly aggressive mucormycete associated with COVID-19-associated mucormycosis (CAM), to germinate and disseminate without exogenous immunosuppression (Almyroudi et al., 2022; Hoenigl et al., 2022). This distinction highlights the unique and profound vulnerability of the diabetic lung and emphasizes that Gas6/Axl-mediated suppression is a major host-side factor that transcends specific fungal species.

The precise molecular triggers for aberrant Gas6 production in the diabetic lung remain incompletely defined; however, several diabetes-associated mechanisms may contribute. Hyperglycemia and the subsequent accumulation of advanced glycation end products (AGEs) induce oxidative stress and activate pro-inflammatory signaling pathways in epithelial cells (Brownlee, 2001). Previous studies in other organs have suggested that high-glucose environments or oxidative stress upregulate Gas6 expression through specific transcription factors, such as Egr-1 or NF-κB, in a cell-type-dependent manner (Nagai et al., 2005). In the present model, the diabetic state may contribute to increased Gas6 production by the airway epithelium, consistent with immunohistochemical findings identifying airway epithelium as a primary source. Resident AMs express substantially higher Axl levels than other pulmonary cell populations and represent likely primary targets of epithelial-derived Gas6. Subsequent ligand-receptor interactions engage a molecular “brake” on macrophage activation via the induction of SOCS family proteins, which downstream suppress NF-κB-mediated transcriptional activity (Rothlin et al., 2007). Consequently, this pre-activated signaling pathway suppresses the chemokine response required during subsequent fungal challenge.

Several limitations should be noted. First, the single high-dose STZ protocol used here does not fully distinguish hyperglycemia from other STZ- or diabetes-associated effects. Validation with insulin-mediated glucose normalization, an alternative low-dose STZ protocol, or a genetic T1D model such as Akita mice would help distinguish these contributions. Our observation that aged, non-STZ-treated mice with mild hyperglycemia also show elevated Gas6 provides preliminary support that Gas6 induction is not STZ-specific, although it does not establish causality. Second, because this study focused on T1D, whether the Gas6/Axl-dependent mechanism extends to type 2 diabetes remains to be determined. Third, although pulmonary fungal burden was quantitatively assessed by morphometric analysis of GMS-positive fungal structures, this approach does not distinguish viable from nonviable fungi and does not capture the total fungal burden throughout the entire lung. Whole-lung fungal CFU or fungal DNA quantification would therefore provide complementary quantitative assessments in future studies.

The therapeutic implications of these findings extend beyond mucormycosis. Because the Gas6/Axl pathway regulates host immune responses rather than fungal-specific processes, targeting this axis may provide broad applicability across diverse pathogens, including fungi, viruses, and bacteria, while simultaneously reducing vulnerability to pathogen-specific resistance mechanisms. Such host-directed approaches may prove particularly valuable in diabetic individuals, who exhibit heightened susceptibility to a broad spectrum of infections, suggesting systemic remodeling of the host immune landscape. In this context, the present findings support a model in which Gas6/Axl-mediated immune modulation represents a convergent mechanism underlying diabetic immune dysfunction, shifting the paradigm from a passive metabolic deficiency toward an active and therapeutically reversible state of immune suppression. Consistent with the translational potential of this concept, BGB324 (bemcentinib), a selective Axl inhibitor, has undergone clinical evaluation in hospitalized patients with COVID-19 (Wilkinson et al., 2020), supporting the feasibility of pharmacological Axl inhibition in human infectious diseases. For high-risk diabetic patients, particularly those with poorly controlled glycemia or diabetic ketoacidosis, pre-emptive targeting of the Gas6/Axl axis may restore immune competence and reduce susceptibility to severe infectious complications.

Finally, these findings underscore the importance of integrated metabolic-immunological perspectives in understanding infection susceptibility. Although glycemic control may reduce factors associated with Gas6 elevation, the precise mechanistic links connecting metabolic dysregulation and immune suppression require further investigation. Therefore, targeting the Gas6/Axl axis may represent a valuable complement to conventional glycemic management by providing a host-directed therapeutic approach to improve defense against severe infections in patients with diabetes.

## Conclusions

In conclusion, we demonstrate that diabetes establishes a pre-existing Gas6/Axl-dependent state of pulmonary innate immune paralysis, which impairs alveolar macrophage chemokine responses, prevents effective neutrophil recruitment, and permits the rapid progression of pulmonary mucormycosis. Our findings identify the Gas6/Axl axis as a mechanistic contributor to impaired pulmonary host defense in this STZ-induced T1D model, and suggest that this pathway may represent a broadly relevant target for restoring pulmonary immunity in patients with diabetes.

## Data Availability

The original contributions presented in the study are included in the article/**Supplementary Material**. Further inquiries can be directed to the corresponding author.

## Supplementary material

Supplementary Figure 1 Neutrophils are essential for host defense against *C. bertholletiae*. **(A)** Experimental scheme for neutrophil depletion using anti-Ly6G antibody (100 μg/mouse, i.p.) 1 day before *Cunninghamella bertholletiae* (Cb) inoculation. **(B)** Survival of control immunoglobulin G (IgG)-treated and anti-Ly6G-treated mice following Cb inoculation (n = 10 mice per group). **(C)** Representative images of cytospin preparations of bronchoalveolar lavage (BAL) fluid. In IgG+*Cb* mice (left), massive neutrophil infiltration and extracellular matrix-like structures (suggestive of neutrophil extracellular traps) are observed. In anti-Ly6G+Cb mice (right), neutrophil recruitment is abolished, and fungal hyphae are prominently visible. **(D)** Absolute number of neutrophils recovered in BAL fluid at 24 h post-inoculation (n = 5 mice per group). **(E)** Representative Grocott’s Methenamine Silver (GMS)-stained lung sections and quantification of fungal burden. Scale bars: 100 μm (upper images); 200 μm (lower images). **(F)** Quantification of GMS-positive areas in lung sections. For each mouse, the percentage of GMS-positive area was calculated as the average of three non-overlapping fields of view (n = 5 mice per group). Data are representative of two independent experiments and are presented as mean ± standard error of the mean. P = 0.0008 by log-rank test **(B)**. ****P < 0.0001 and ***P < 0.001 by Mann–Whitney test **(D, F)**.
